# Identification of QTL associated with resistance to Phytophthora fruit rot in cucumber (*Cucumis sativus* L.)

**DOI:** 10.3389/fpls.2023.1281755

**Published:** 2023-11-15

**Authors:** Ying-Chen Lin, Ben N. Mansfeld, Xuemei Tang, Marivi Colle, Feifan Chen, Yiqun Weng, Zhangjun Fei, Rebecca Grumet

**Affiliations:** ^1^ Department of Horticulture, Graduate Program in Plant Breeding, Genetics and Biotechnology, Michigan State University, East Lansing, MI, United States; ^2^ Boyce Thompson Institute, Cornell University, Ithaca, NY, United States; ^3^ Department of Plant and Agroecosystem Sciences, University of Wisconsin, Madison, WI, United States; ^4^ Vegetable Crops Research Unit, United States Department of Agriculture-Agriculture Research Service (USDA-ARS), Madison, WI, United States; ^5^ Robert W. Holley Center for Agriculture and Health, United States Department of Agriculture-Agriculture Research Service (USDA-ARS), Ithaca, NY, United States

**Keywords:** *Phytophthora capsici*, disease resistance, age-related resistance, QTL-seq, GWAS, XP-GWAS, cucumber core collection

## Abstract

Phytophthora fruit rot (PFR) caused by the soilborne oomycete pathogen, *Phytophthora capsici*, can cause severe yield loss in cucumber. With no resistant variety available, genetic resources are needed to develop resistant varieties. The goal of this work was to identify quantitative trait loci (QTL) associated with resistance to PFR using multiple genomic approaches and populations. Two types of resistances have been identified: age-related resistance (ARR) and young fruit resistance. ARR occurs at 12-16 days post pollination (dpp), coinciding with the end of exponential fruit growth. A major QTL for ARR was discovered on chromosome 3 and a candidate gene identified based on comparative transcriptomic analysis. Young fruit resistance, which is observed during the state of rapid fruit growth prior to commercial harvest, is a quantitative trait for which multiple QTL were identified. The largest effect QTL, *qPFR5.1*, located on chromosome 5 was fine mapped to a 1-Mb region. Genome-wide association studies (GWAS) and extreme-phenotype genome-wide association study (XP-GWAS) for young fruit resistance were also performed on a cucumber core collection representing > 96% of the genetic diversity of the USDA cucumber germplasm. Several SNPs overlapped with the QTL identified from QTL-seq analysis on biparental populations. In addition, novel SNPs associated with the resistance were identified from the germplasm. The resistant alleles were found mostly in accessions from India and South Asia, the center of diversity for cucumber. The results from this work can be applied to future disease resistance studies and marker-assisted selection in breeding programs.

## 1 Introduction

Cucumber production in the United States primarily serves two markets: slicing (fresh) and pickling (processing). Pickling cucumber production using primarily the once-over machine harvesting system in the Midwestern U.S. is severely impacted by Phytophthora fruit rot caused by the soil-borne oomycete, *Phytophthora capsici* (Hausbeck and Lamour, 2004; Savory et al., 2011). The pathogen primarily infects cucumber fruits, especially young, rapidly growing fruits, while other plant parts such as leaves and vines remain intact (Gevens et al., 2006). The cucumber plants grown for processing are planted on bare ground in high density to facilitate machine harvest. The fruits, which are beneath the vegetative canopy, are in direct contact with the infested soil and the high density of foliage creates a moist environment ideal for the growth and spread of *P. capsici* (Ando and Grumet, 2006; Ando et al., 2007). The pathogen releases flagellate zoospores from sporangia that are mobile in water and can easily spread within or between fields through irrigation or rainwater (Granke et al., 2012). The symptoms start with water-soaked lesions and tissue collapse, followed by the growth of white mycelia and sporangia on the fruit surfaces (Quesada-Ocampo et al., 2023). Currently, growers rely on a combination of exclusion, cultural practices for management, and chemical control strategies to reduce outbreaks of *P. capsici* (Sanogo et al., 2022; Quesada-Ocampo et al., 2023). However, *P. capsici* can readily develop resistance to chemical controls and several commonly used fungicides such as metalaxyl and mefenoxam have been shown to be ineffective against the pathogen (Kousik et al., 2017; Quesada-Ocampo et al., 2023). Growing resistant varieties could reduce the use of fungicides, which should lead to lower cost and less potential environmental or health hazards. However, the complex nature and genetic variation in both host and pathogen hamper the development of resistant commercial cultivars. In cucumber, sources of resistance were discovered (Gevens et al., 2006; Colle et al., 2014), but are yet to be introgressed into commercial varieties.

The effort of searching for resistance to *P. capsici* initially led to the discovery of age-related resistance (ARR) (Gevens et al., 2006; Ando et al., 2009). The fruits from accessions expressing ARR (ARR+) are susceptible at early fruit development stages, then gradually become resistant as fruits develop (Ando et al., 2015). The transition begins at ~12 days post pollination (dpp), toward the end of exponential fruit growth, and was demonstrated to be associated with the fruit peel (Ando et al., 2012; Ando et al., 2015). Preformed biochemical defenses and metabolites that are developmentally regulated were found to be associated with ARR, these include enzymes producing defense related compounds such as reactive oxygen species and terpenoid glycosides (Mansfeld et al., 2017). Resistant-aged fruit also appear to be uniquely able to sense the presence of *P. caps*ici zoospores, as evident by a spike in defense response genes as early as 2 hours post inoculation. This corresponds with observable death of zoospores early as in 4 hour-post-inoculation in fruits that exhibit ARR (Mansfeld et al., 2020).

While ARR is a largely effective form of resistance, cucumber fruits are usually harvested and consumed during mid- to late- exponential growth (approximately 8-12 dpp), prior to the transition to ARR. Hence, cucumber growers will still suffer yield loss due to *P. capsici* even if the cultivars exhibit ARR. Therefore, resistance expressed before the harvest age is desired to alleviate the yield loss caused by the pathogen. To search for young fruit resistance, Colle et al. (2014) surveyed the U.S. cucumber plant introduction (PI) by testing young cucumber fruits (~5-7 dpp). Three accessions with low disease scores were potential sources of young fruit resistance. One of them, PI 109483 from Türkiye, exhibited stable resistance in the following generations of selfing. The resulting S_6_ progeny was released as a breeding line MSU 109483-53 (Grumet and Colle, 2017). The breeding line was later used for doubled haploid (DH) production, and the DH line ‘A4-3’, which shows delayed and reduced symptoms and slower rate of pathogen growth (Zhang et al., 2021), was used to study young fruit resistance.

The objectives of this work were to identify genetic loci associated with both young fruit resistance and ARR and to develop molecular markers for future breeding efforts. Multiple genetic and genomic approaches, including bulk segregant analyses, fine mapping, transcriptome analyses and genome wide association studies identified several loci in association with the resistance traits. Association analyses identified several SNPs that overlapped with QTL identified from QTL-seq analysis as well as novel SNPs and potential sources of resistance.

## 2 Materials and methods

### 2.1 Plant materials

#### 2.1.1 Biparental mapping

The source of young fruit resistance was MSU109483-53 (Grumet and Colle, 2017), a breeding line obtained through a series of pure line selections from PI 109483, a landrace collected from Türkiye. Seed from MSU109483-53 was used for doubled haploid (DH) production via *in vivo-*induced parthenogenic embryo culture generously performed by Rijk Zwaan (De Lier, Netherlands). The DH line ‘A4-3’ was crossed with the susceptible parent, ‘Gy14’, an American type pickling cucumber, which has been broadly used in research and breeding programs and for which a high-quality reference genome is available (http://cucurbitgenomics.org/v2/). ‘A4-3’ also was crossed with an American fresh market cucumber, ‘Poinsett 76’, which exhibits ARR (Mansfeld et al., 2020), to provide a second population for QTL verification. To map ARR, ‘Gy14’ (ARR-) was crossed with ‘Poinsett 76’ (ARR+) and resultant F_1_ seed was sent for DH production (Rijk Zwaan, De Lier, Netherlands).

#### 2.1.2 Cucumber core collection

The cucumber core collection was selected based on genotyping-by-sequencing (GBS) data of United States National Plant Germplasm System (NPGS) collection (Wang et al., 2018). To reduce heterozygosity and heterogeneity within the accessions, individuals from each accession were self-pollinated for 2 or 3 generations. The self-pollinated core collection lines were re-sequenced at 30-40× coverage and used to call SNPs as described by Yu et al. (2023) (http://cucurbitgenomics.org/v2/). The core collection lines were grown in the field from 2019-2022 with three plants per accession. The accessions that were tested are listed in **Supplementary Table 1**. The number of accessions planted each year varied depending on seed availability; each accession has 1-4 years of phenotypic data.

### 2.2 Growth conditions

Seeds for all experiments were sown in the greenhouse or growth room. Seedlings were transplanted to the greenhouse or field at the two true-leaf stage. No fungicide was applied after the onset of flowering in either the greenhouse or field to ensure that fungicide residue was not present on the fruit surface to interfere with phenotyping.

#### 2.2.1 Greenhouse

Seedlings were transplanted to 1.5-gallon pots with Suremix Perlite soil medium and grown in the Michigan State University Plant Science Greenhouse Complex. The plants were fertigated twice a day (44 ppm nitrogen of Peters Professional 20-20-20 General Purpose; Scotts, Marysville, OH). LED lights were used to provide 16-hour photoperiod. Pest and disease management was based on general practice in the greenhouse using a combination of chemical and biological controls. For young fruit experiments, bumble bees (Koppert Biological Systems, Inc., Howell, MI) were introduced at week 4-5 for pollination. For ARR experiments, flowers were hand-pollinated; a single fruit was set per plant to prevent developmental effects of competition among fruits. All ARR experiments were grown in the greenhouse.

#### 2.2.2 Field

Plants were grown at the Michigan State University Horticulture Teaching and Research Center (HTRC). Prior to transplanting, 300 lbs/acre of 19-19-19 fertilizer were applied to the field, and irrigation provided as needed throughout the season. To minimize contamination with other pathogens and avoid injury resulting from washing soil from the fruit, plants were grown on raised black plastic mulch and trellised using T-posts and trellis netting. The space between plants was 0.45-0.6 m, depending on the field design each year. Pollination was facilitated by honeybees.

### 2.3 Screening for response to *P. capsici*

#### 2.3.1 Fruit harvesting and handling

To provide uniform, high-inoculum pressure and optimal environmental conditions for disease development, fruits grown in both the greenhouse and field were harvested at the desired stage of development and brought into the laboratory for disease screening. For young fruit experiments, fruit were harvested at early exponential growth stage, 5-7 dpp (~7-10 cm long). Harvests were performed 2-3 times a week to provide 30-60 fruit/accession for the core collection, or 10-30 fruit/plant for biparental segregating populations. For ARR experiments, fruit were harvested at 16-18 dpp. Fruit from the field were rinsed with distilled water to remove soil and debris, sanitized by soaking in 1% bleach for one minute, and rinsed with distilled water thoroughly to remove bleach residue. Greenhouse fruit were rinsed with distilled water. Clean, dry fruits were placed in covered plastic trays lined with wet paper towel on the sides to maintain high humidity for pathogen growth as described by Gevens et al. (2006). Trays were incubated at 25-26 C under constant light.

#### 2.3.2 Pathogen inoculation and phenotyping

**Figure 1:**
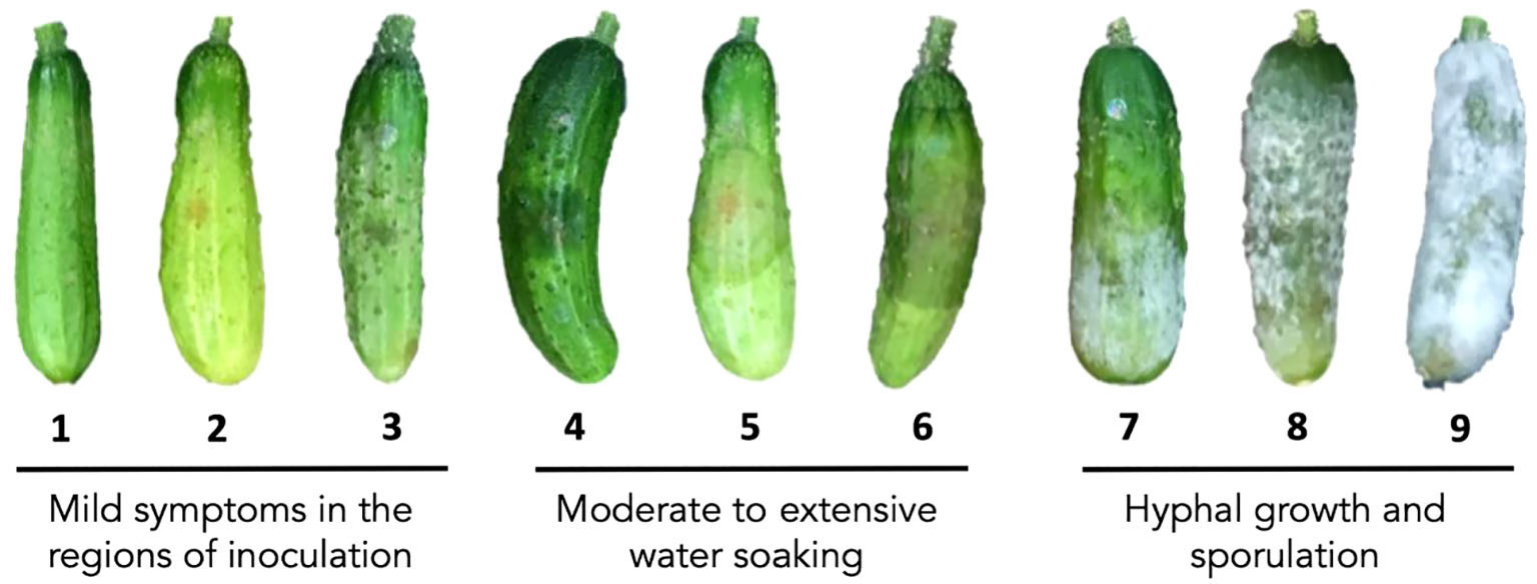
Illustration of the 9-point disease scoring scale of *Phytophthora capsici* infection on cucumber fruit. Ratings 1- 3: no or minor symptoms limited to inoculation sites; ratings 4-6: levels of water soaking and necrosis; ratings 7-9: different levels of hyphal growth and sporulation.

The *P. capsici* isolates, Bartley’s 1, OP97, or NY-0644-RFP (Dunn et al., 2013), were cultured on V8 agar media as described in Gevens et al. (2006). After seven days, the plate was flooded with 6-7 mL sterile distilled water to stimulate zoospore production. The concentration of resuspended zoospore was measured using a Countess™ automated cell counter (Thermo Fisher Scientific, Waltham, MA). For young fruit experiments, zoospore suspensions of Bartley’s 1 were diluted to 1×10^4^ zoospore/mL. Two 30 μL droplets were applied to the surface of each fruit as described by Colle et al. (2014). Inoculated cucumber fruits were photographed and scored at 5 days post inoculation (dpi) based on the disease rating scale shown in **Figure 1**. Ratings of 1-3 indicate mild symptoms limited the region of inoculation, 4-6 moderate to extensive water soaking, and 7-9 visible hyphal growth and sporulation. Symptoms were scored at both of the inoculated sites on each fruit; the score for an individual fruit was the mean of the two sites. The disease rating for a plant or line was the average of all fruit over all harvests for a given experiment. For the ARR experiments, OP97 or NY-0644-RFP zoospore suspensions were diluted to 1×10^5^ zoospores/mL. Disease symptoms were monitored daily for 7-10 days and rated using either a 1-9 disease rating scale as above (F_2_ experiments) or a 0-5 point disease score (0 – no symptoms, 5 – severe sporulation) (DH experiments). Fruits from the DH population were inoculated with 12 equally spaced 30 μL droplets. At 7 dpi, each fruit was assigned the rank of the most susceptible inoculation site.

### 2.4 QTL-seq analysis

#### 2.4.1 Young fruit resistance

An F_2_ population (n=362) from the ‘Gy14’ × ‘A4-3’ was screened in 2018 in the field. Leaf tissue (~50mg) was collected from each seedling prior to transplanting to the field, freeze-dried, and ground for DNA extraction. DNA was extracted from the parental lines and each plant selected for the bulks using the Kingfisher DNA extraction robot and Mag-Bind® Plant DNA DS 96 Kit (Omega Bio-tek, Norcross, GA) as described in Wang et al. (2018). The genomic DNA was quantified using PicoGreen (Invitrogen™, Waltham, MA). Equal amounts of DNA of the selected individuals for each bulk (n=19) were mixed for sequencing. Sequencing was performed at the Research Technology Support Facility at Michigan State University. Three libraries (resistant bulk, susceptible bulk, and ‘A4-3’) were prepared using Illumina TruSeq Nano DNA Library Preparation Kit and sequenced using paired-end sequencing of 150 bp on an Illumina HiSeq 4000 platform. After removing low-quality reads and sequencing adaptors using Trimmomatic v. 0.33 (Bolger et al., 2014), the reads from each bulk were aligned to the ‘Gy14’ reference genome v2.0 (http://cucurbitgenomics.org/v2/; Yu et al., 2023) using BWA-MEM (v0.7.8) (Li, 2013) with default parameters. Sequencing duplicates were marked using PicardTools (https://broadinstitute.github.io/picard/, v2.7.1) and the Genome Analysis Toolkit (GATK; v3.6) best practice pipeline was used for SNP calling (McKenna et al., 2010; DePristo et al., 2011; Van der Auwera et al., 2013). QTL-seq analysis (Takagi et al., 2013) was performed using the R package, QTLseqr (Mansfeld and Grumet, 2018). SNP filtering criteria in QTLseqr was set for “minimum depth ≥ 50, maximum depth ≤ 100, GQ ≥ 99, depth difference ≥ 20, minimum sample depth ≥ 20,” which left 587,178 SNPs for further analysis. Delta SNP-index and G’-value (Magwene et al., 2011) were calculated using a sliding window size of 1Mb, where the 95% and 99% confidence intervals were calculated with 10,000 iteration in QTL-seq analysis, while filter method “deltaSNP” at the threshold of 0.1 in G’ analysis.

#### 2.4.2 ARR

Progeny of 79 DH lines (5 plants/line) along with both parents and F_1_ seed were grown in the greenhouse in a replicated block design. In a separate experiment, 92 F_2_ plants of the ‘Gy14’ × ‘Poinsett 76’ cross were grown in the greenhouse; plants with the 15 highest and lowest disease scores were selected for the two bulks respectively. DNA extraction was as described above. After quantitation, all libraries were pooled in equimolar amounts and loaded on one lane of an Illumina HiSeq 2500 High Output flow cell (v2) and sequenced in a 2 ×150bp paired end format. Reads were cleaned and adaptor sequences were removed using Trimmomatic v. 0.33 (Bolger et al., 2014). QTL-seq analysis was performed as previously described with the following settings (F2/DH): refAlleleFreq = 0.1/0.1, minTotalDepth = 20/30, maxTotalDepth = 50/100, depthDifference = 10/30, minGQ = 30/30, minSampleDepth = 10/15. A window size of 2 Mb was used for smoothing Δ(SNP-index) values.

### 2.5 Verifying and narrowing the genomic regions

#### 2.5.1 Young fruit

A second F_2_ population (‘Gy14’× ‘A4-3’; n=752) was used to verify the QTL regions and develop RIL and F_3_ populations. Polymorphic SNPs flanking and within the QTL region were identified for KASP marker design and ordered from Sigma-Aldrich (St. Louis, MO). The 5 μL reaction mixture contained 2.5 μL of DNA (at 10 ng/μL), 2.5 μL of 2 × KASP Master Mix (LGC Biosearch Technologies, Hoddesdon, UK; 3CR Bioscience, Essex, UK), and 0.07 μL of primer mix. The cycling conditions were as follows: 94°C for 15 min followed by 10 touchdown cycles at 94°C for 20 s and 65°C for 60 s (decrease 0.6°C per cycle), then 38 amplification cycles of 94°C for 20 s and 55°C for 60 s, finally with 37°C for 10 s. Thermocycling and fluorescence readings were performed using a FX384 Real-Time thermal cycler (BioRad, Hercules, CA), where allele calls were determined using the CFX manager software (v.3.1). To narrow the genomic region, homozygous recombinant individuals (i.e., homozygous for the ‘Gy14’ allele at one and homozygous for the ‘A4-3’ allele at the other end) were selected and self-pollinated for 4-5 generations to develop a RIL population. Individuals that were partially heterozygous (heterozygous at one end, and homozygous for the ‘Gy14’ or ‘A4-3’ allele at the other end) were selfed to produce F_3_ families. KASP markers were designed at approximately every 0.5 Mb within the QTL region. The sequences of KASP markers and targeted SNP locations are in **Supplementary Table 2**. RIL families were grown in the field in 2020 with 30-60 fruits tested per line. In 2022, 99 homozygous recombinant individuals from 33 F_3_ families were grown in the greenhouse. Cuttings were also taken from each plant and transplanted to the field. Fruit were harvested from both sets of plants and disease screening was performed as described above.

#### 2.5.2 ARR

To verify the ARR QTL, KASP markers flanking the QTL were used to genotype 768 F_2_ seedlings of ‘Gy14’ × ‘Poinsett 76’ as described above. Individuals homozygous for ‘Gy14’ or ‘Poinsett76’ alleles within the QTL region were self-pollinated to produce F_4_ lines. Plants from 14 F_4_ lines were grown in the greenhouse in a randomized complete block trial (5 plants/line). Phenotyping was performed as described above.

### 2.6 RNA-seq analysis

#### 2.6.1 Sample collection and RNA extraction

Flowers of plants from the ARR parental lines (‘Poinsett 76’, ‘Gy14’) were hand pollinated so that 8 and 16 dpp fruit were harvested on the same day. Three fruit (biological replicates) were collected for each age and genotype. Uninoculated fruit peels were collected from 8 and 16 dpp fruit using a vegetable peeler and immediately frozen in liquid nitrogen. Samples were ground using a mortar and pestle in liquid nitrogen. RNA extraction was performed using the MagMAX Plant RNA Isolation Kit protocol (Thermo Fisher, Waltham, MA) as described in Mansfeld et al. (2020). Assessment of RNA concentration and quality was performed as described in Rett-Cadman et al. (2019). All samples had a minimum RNA quality score of 8.

#### 2.6.2 TruSeq library preparation and sequencing

Libraries were prepared at Michigan State University’s Research Technology Support Facility, using the Illumina TruSeq Stranded mRNA Library Preparation Kit on a Sciclone G3 robot following manufacturer’s recommendations. An additional cleanup with 0.8 × AmpureXP magnetic beads was performed after completion of library preparation. Quality control and quantification of completed libraries were performed using a combination of Qubit dsDNA HS and Advanced Analytical Fragment Analyzer High Sensitivity DNA assays. The libraries were divided into two pools of 15 libraries each. Pools were quantified using the Kapa Biosystems Illumina Library Quantification qPCR kit. Each pool was loaded onto one lane of an Illumina HiSeq 4000 flow cell and sequencing was performed in a 1 × 50 bp single read format using HiSeq 4000 SBS reagents. Base calling was done by Illumina Real Time Analysis (RTA) v2.7.7 and output of RTA was demultiplexed and converted to FastQ format with Illumina Bcl2fastq v2.19.1.

#### 2.6.3 Differential expression analysis

Reads were cleaned, and adaptor sequences were removed using Trimmomatic v. 0.34 (Bolger et al., 2014) with the following settings: LEADING:3 TRAILING:3 SLIDINGWINDOW:4:15 MINLEN:35. Quality control was performed using FastQC (http://www.bioinformatics.bbsrc.ac.uk/projects/fastqc). A cucumber transcriptome fasta file was made from the ‘Chinese Long’ (v2) (Huang et al., 2009; Li et al., 2011) genome using the gffread function from the cufflinks software package (Trapnell et al., 2010) and high-quality reads were then quasi-mapped to the transcriptome using Salmon v. 0.9.1 (Patro et al., 2017) with default settings. Read quantification data was imported into R using the tximport R package (Soneson et al., 2015) and differential expression analysis was performed using DEseq2 (Love et al., 2014) with log-fold-change-shrinkage. Age and genotype were combined into a single factor for differential expression analysis and contrasts between the four conditions (‘Poinsett 76’ 8 dpp, ‘Poinsett 76’ 16 dpp, ‘Gy14’ 8 dpp, ‘Gy14’ 16 dpp) were performed. Differentially expressed genes were called significant using an adjusted p-value (Benjamini-Hochberg adjustment; false discovery rate) of less than 5% and an expression change of greater than two-fold was used to define biological significance.

### 2.7 Association analysis

#### 2.7.1 GWAS

The SNP data of the core collection was downloaded from CucGenDBv2 (Yu et al., 2023). SNPs were filtered using BCFtools (Danecek et al., 2021) and GATK (Van der Auwera et al., 2013) with the following criteria: bi-allelic, GQ scores >20, maximum read depth within two standard deviations of the mean read depth, minor allele frequency > 0.1, missing rate <20%, resulting in 1,168,270 SNPs for association analysis. Marker-trait association analyses were performed using best linear unbiased estimates (BLUEs). BLUEs were calculated using the R package lme4. (Bates et al., 2015) Association analysis was performed using GAPIT 3.0 (Genome Association and Prediction Integrated Tool) with MLM, FarmCPU, BLINK, and MLMM models implemented within the software (Wang and Zhang, 2021). The significance threshold was calculated based on Bonferroni correction (p-value/N, N= number of SNPs used in the analysis), where the thresholds of adjusted p values of 0.05 and 0.01 corresponded to -log10(p) values of 7.368 and 8.06, respectively.

#### 2.7.2 XP-GWAS

The 29 most resistant and 29 most susceptible accessions were selected based on 2019-2021 phenotypic data and grown in the field in a randomized complete block design with three blocks in 2022. A random bulk of 29 accessions was selected from the full core population (**Supplementary Table 1**). XP-GWAS analysis was performed as described in Yang et al. (2015). In brief, reference and alternative allele depths at each SNP site were extracted from the core resequencing data and calculated for each bulk. The input data was then computed using the R package, XP-GWAS, with the depth filter set at 500, which ended with 3,444,143 SNPs for the analysis. The 5% false discovery rate (FDR) and the threshold of p = 0.05 with Bonferroni correction were calculated to detect significant SNPs.

## 3 Results

### 3.1 Identification of QTL for young fruit resistance

#### 3.1.1 QTL-seq analysis

**Figure 2:**
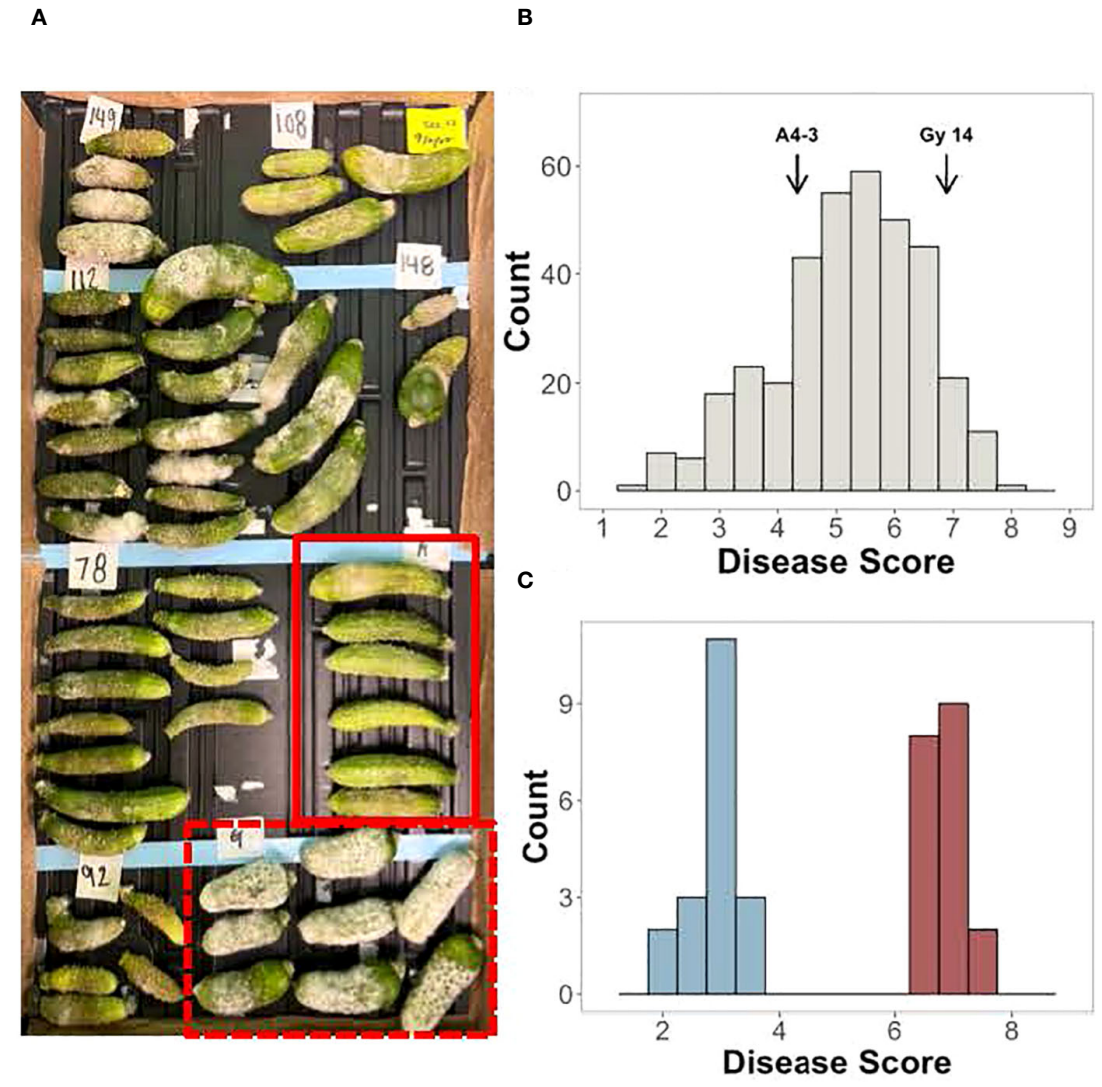
Screening of young fruit from the F_2_ population of ‘Gy14’ × ‘A4-3’ for response to inoculation with *P. capsici*. Fruits were harvested at 5-7 days post pollination (dpp). **(A)** Example of disease screening; fruits were photographed at 5 days post inoculation (dpi). Red box, A4-3; Dashed box, ‘Gy14’. **(B)** Disease score distribution of the F_2_ population. Scores are the average of 10-20 fruit from each F_2_ plant; fruit were scored at 5 dpi. **(C)** F_2_ individuals selected for the resistant and susceptible bulks. Disease scores are the average of all four harvests.

Screening for young fruit resistance to *P. capsici* was performed on an F_2_ population (n=362) derived from the cross between the susceptible pickling cucumber breeding line, ‘Gy14’, and the doubled haploid line, ‘A4-3’, which shows reduced and delayed symptom development in response to inoculation (**Figure 2A**). Three harvests were performed with 10-20 young fruits (5-7 dpp; ~7-10 cm long) sampled from each F_2_ plant. The normally distributed disease scores suggested that young fruit resistance is a quantitative trait controlled by multiple loci (**Figure 2B**). To verify phenotyping of plants to be selected for the resistant and susceptible bulk populations, the 30 highest and lowest scoring individuals from the first three harvests were harvested an additional time. Based on the four harvests, 19 plants which had consistent phenotypes were selected for the resistant and susceptible bulks, respectively (**Figure 2C**).

**Figure 3:**
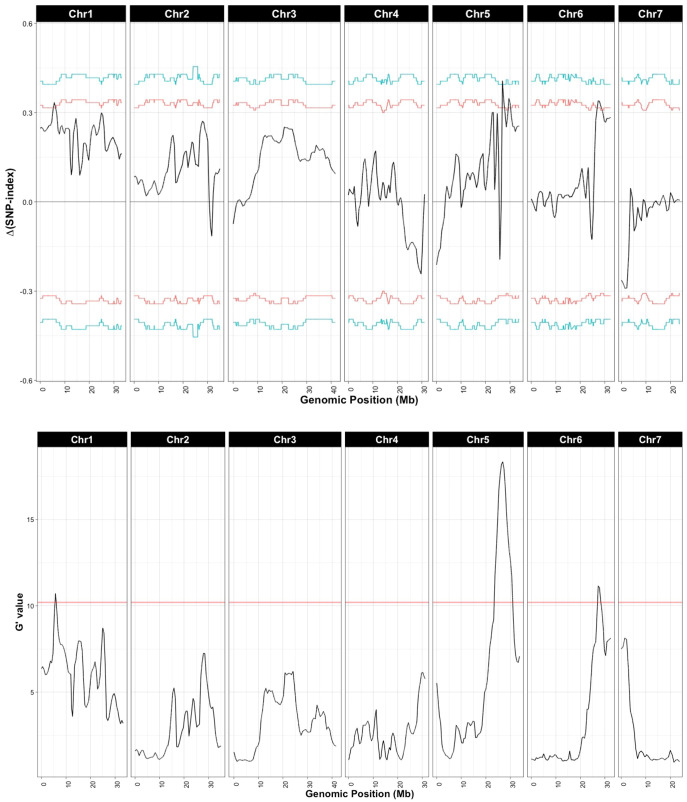
QTL associated with young fruit resistance as identified by QTL-seq. Δ(SNP-index) and G’ were calculated with a window size of 1 Mb. Horizontal lines in Δ(SNP-index) represent confidence thresholds of 95% (red) and 99% (blue); for G’ the threshold for false discovery is 0.01 (blue line). Allele frequencies for the resistant and susceptible bulks are provided in **Supplementary Figure 1**.

Sequencing of the bulk populations generated ~77 million reads for each bulk with > 60× coverage of the cucumber genome. After processing and filtering, 558,625 SNPs were available for QTL-seq analysis, which identified QTL on chromosomes 1, 5, and 6 (**Figure 3**; **Table 1**). The most significant QTL was located on chromosome 5.

**Table 1:** QTL identified from QTL-seq in the ‘Gy14’ x ‘A4-3’ F_2_ population.

Chromosome	QTL-seq		G’	
	Location (Mb)^1^	Length	Location (Mb)	Length
1	5.35-6.27	0.92	5.53-6.00	0.47
5	26.87-27.71	0.84	23.40-30.94	7.53
5	29.24-30.19	0.95	–	–
6	26.92-28.95	2.03	27.04-28.38	1.34

^1^Genomic locations are according to Gy14 v. 2.1 (CuGenDB v.2; http://cucurbitgenomics.org/v2/).

#### 3.1.2 QTL validation and narrowing the genomic region of *qPFR5.1*

**Figure 4:**
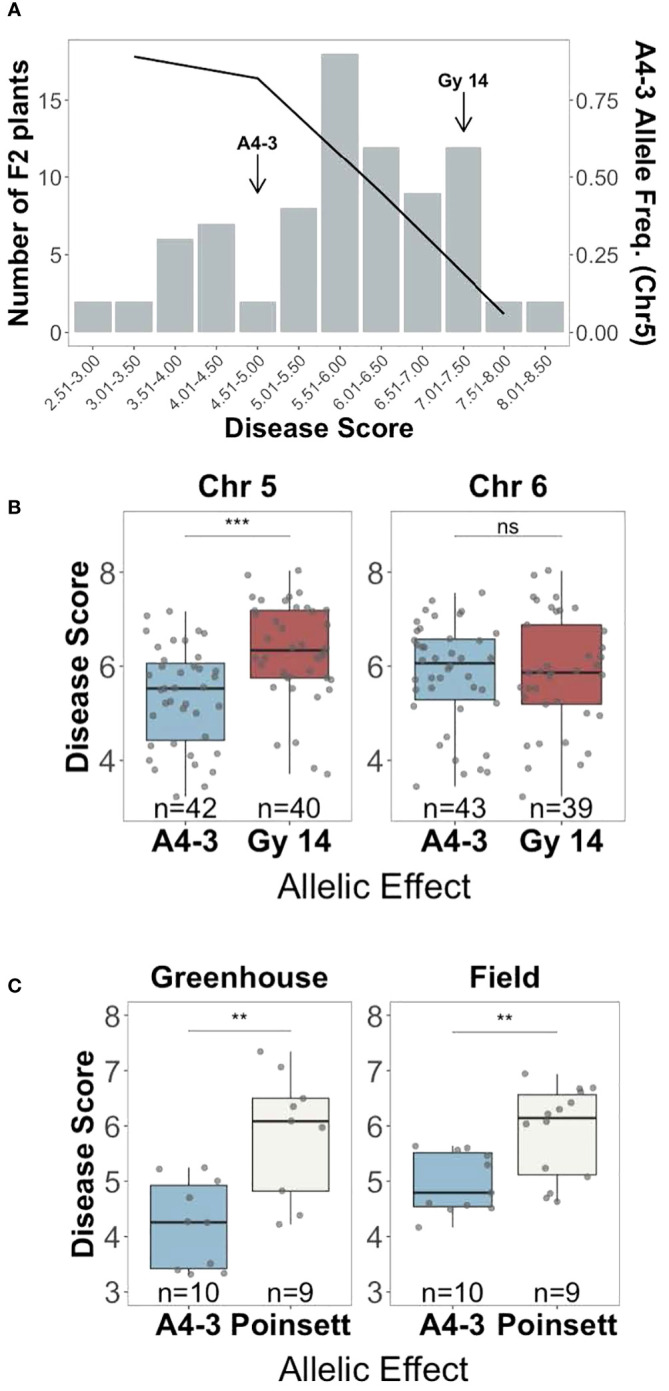
Verification of the allelic effect of QTL for young fruit resistance identified by QTL-seq analysis. **(A)** Disease distribution at 5 days post inoculation for the selected F_2_ plants (‘Gy 14’ × ‘A4-3’) homozygous for the ‘Gy14’ or ‘DH4-3’ alleles (n=82) (bar graph), and the frequency of the resistant ‘A4-3’ allele on chromosome 5 (black line). Each F_2_ value is the mean of 10-30 fruit/plant. **(B)** Disease scores of F_2_ individuals of ‘Gy14’ (pickling cucumber) ‘A4-3’ possessing either the ‘A4-3’ or ‘Gy14’ allele at the predicted QTL at chromosomes 5 (*qPFR5.1*) and 6. **(C)** F_3_ families of ‘Poinsett 76’ (fresh market cucumber) ‘A4-3’ possessing either the ‘A4-3’ or ‘Poinsett’ allele at *qPFR5.1*. Each point is the mean of >20 fruits/family from the greenhouse and >50 fruits/family from the field. Ns, non-significant; **P < 0.01; ***P < 0.001.

To validate the regions identified by QTL-seq, a second F_2_ population (n=752) was genotyped using KASP markers flanking the QTL. Individuals homozygous for either susceptible ‘Gy14’ (S) or resistant ‘A4-3’ (R) alleles on chromosome 5 and/or 6 were selected, providing four allelic combinations (chr5-chr6: Gy-Gy, Gy-A4-3, A4-3-Gy, and A4-3-A4-3). Presence of the ‘A4-3’ allele on chromosome 5 was associated with resistance with a frequency of 0.89 in the most resistant plants (disease score < 4) progressively dropping to 0.06 in the most susceptible plants (disease score > 7) (**Figure 4A**). Individuals with ‘A4-3’ alleles at chromosome 5 showed significantly lower disease score compared to those with ‘Gy14’ allele (**Figure 4B**). No significant allelic effect was observed for the QTL on chromosome 6 (**Figure 4B**). The allelic effect of the QTL on chromosome 1 was not verified.

The QTL on chromosome 5, named *qPFR5.1 (Phytophthora fruit rot 5.1)*, was tested in a second genetic background, ‘Poinsett 76’, a North American fresh market cucumber. F_2_ individuals from ‘Poinsett 76’ × ‘A4-3’ (n=768) were genotyped with flanking markers for *qPFR5.1*, and individuals homozygous for either ‘Poinsett 76’ or ‘A4-3’ in that region were self-pollinated. Plants from the resulting 25 F_3_ families were grown in the greenhouse and field and young fruit were harvested for inoculation. Consistent with the RIL population, the ‘A4-3’ allele showed strong association with resistance in the greenhouse and field, respectively), further confirming the effect of *qPFR5.1* (**Figure 4C**).

**Figure 5:**
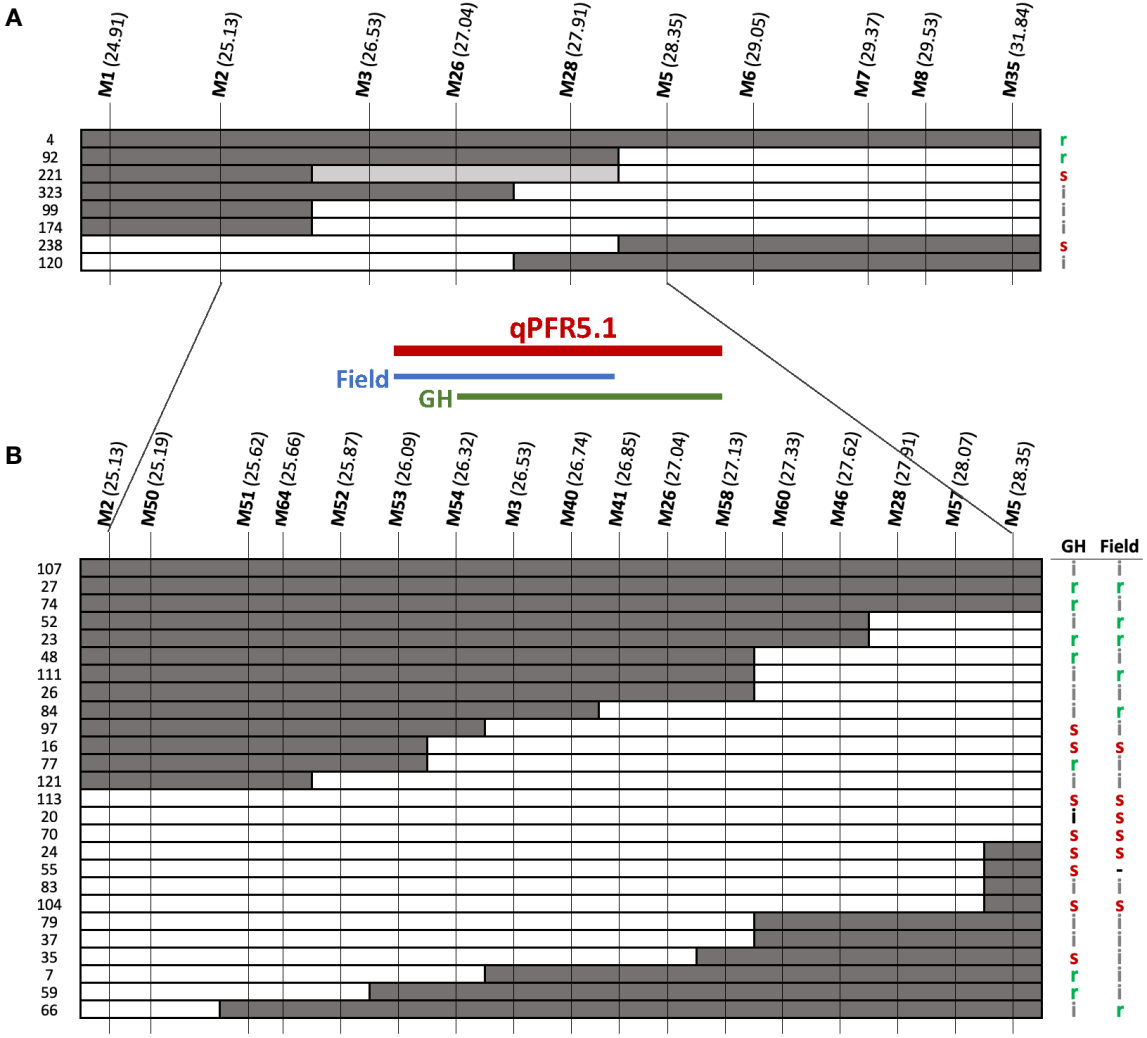
Fine mapping of *qPFR5.1* in **(A)** RIL population and **(B)** F_3_ families. Dark, white, grey bars represent ‘A4-3’, ‘Gy14’, and heterozygous alleles, respectively. Letters on the right indicate phenotypes of each line/family: r, resistant (score < 4.5); s, susceptible (score > 6.0); i, intermediate (scores 4.5 - 6.0). Genomic locations shown in parentheses for each marker (Mb) as per Gy14 v. 2.1 (CuGenDB v.2; http://cucurbitgenomics.org/v2/).

*qPFR5.1* as identified by QTL-seq and G’ spanned ~7Mb on chromosome 5. To facilitate fine mapping, we tested a recombinant inbred line (RIL) population and F_3_ families selected for recombination within the QTL region. F_2_ plants that were homozygous recombinant at *qPFR5.1* (*i.e*., homozygous for the ‘Gy14’ allele at one end and homozygous for the ‘A4-3’ allele at the other end) were self-pollinated to the F_4_ and F_5_ generations; nine resulting RILs were grown in the field in 2020 with 30-60 fruits tested per line. Each plant was also genotyped with KASP markers at approximately 0.5 Mb intervals within the QTL region (**Figure 5A**). Based on genotypic and phenotypic data, the region was narrowed to 3.22 Mb between markers M2 and M5 (25.17-28.39 Mb).

To further refine the QTL, an additional 768 F_2_ plants were genotyped and 178 partially homozygous F_2_ individuals (i.e., heterozygous at one end and homozygous for ‘A4-3’ or ‘Gy14’ alleles at the other end) were self-pollinated. Twelve F_3_ families were selected for each of the four genotypic combinations (heterozygous-’Gy14’, ‘Gy14’-heterozygous, heterozygous-’A4-3’, and ‘A4-3’-heterozygous) and 16 individuals per family were genotyped. Of those, 99 homozygous recombinant individuals from 33 families were transplanted to the greenhouse. Cuttings were also taken from each plant and transplanted to the field for testing in a second environment. Additional markers were designed between M2 and M5 (**Figure 5B**). The QTL identified from F_3_ families was located between M53-M58 (26.09-27.13 Mb; 1.04 Mb) with a slight difference between the two seasons tested; M54-M58 (26.32-27.13 Mb) in the greenhouse, and M53-M41 (26.01-26.85 Mb) in the field.

### 3.2 Association analysis of resistance to *P. capsici*

#### 3.2.1 GWAS of the core collection

**Figure 6:**
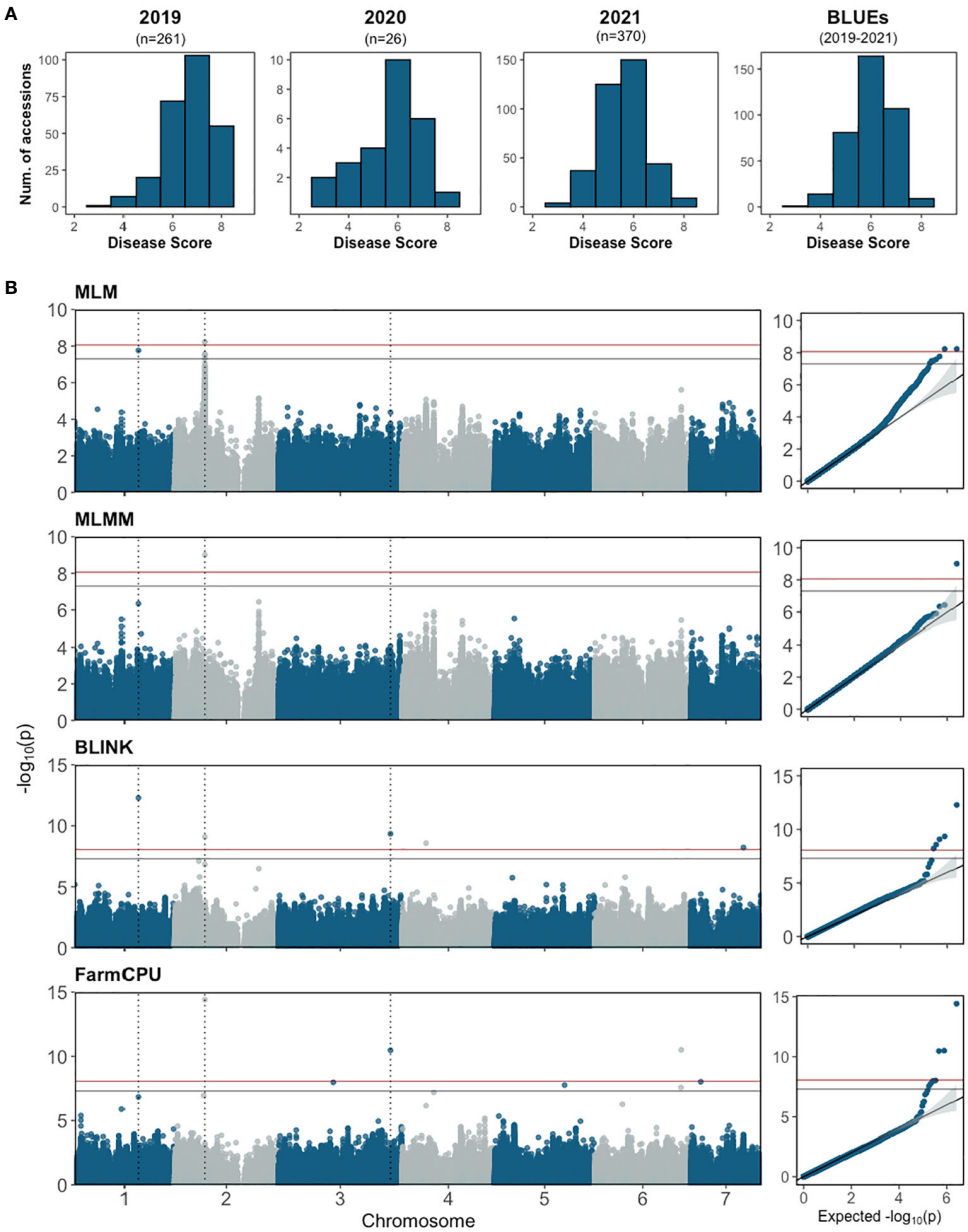
**(A)** Disease score distribution for young fruit resistance to *Phytophthora capsici* from the cucumber core collection and BLUE distribution of combined data 2019-2021. The score for each accession is the mean of 30-50 fruits. **(B)** Manhattan plots and quantile–quantile plots of the genome-wide association study analyses for young fruit resistance in the cucumber core population. The horizontal blue and red lines represent significance thresholds of Bonferroni -corrected *P* values of 0.05 and 0.01, respectively. The dotted vertical lines show the locations of SNPs that were significant in at least two models.

The re-sequenced cucumber core collection, consisting of 388 accessions along with several breeding lines with important agronomic traits, represents >96% of genetic diversity in the U.S. NPGS (Wang et al., 2018; Yu et al., 2023). The collection was planted in the field and fruits were tested from 2019 to 2021. The number of accessions grown each year varied depending on seed availability. Phenotypic data was collected from 370 accessions with 1-3 years of disease scores per accession. The normally distributed disease score of the core collection further affirmed that young fruit resistance is a quantitative trait (**Figure 6A**); the correlation between years ranged from 0.48-0.80.

GWAS analysis was performed using BLUE values calculated from disease scores from 2019-2021 with one single-locus model (MLM) and three multi-locus models (FarmCPU, BLINK and MLMM) (**Figure 6B**; **Table 2**). A total of 11 SNPs were identified from the different models: seven in FarmCPU, five in BLINK, one in MLMM, and five in MLM. The phenotype variance explained (PVE) of the SNPs ranged from 0.38-24.49%. Several significant SNPs were identified in at least two models. S1_21117743 (A/G) was significant in BLINK and MLM models with PVE of 9.01% and 7.74%, respectively. S2_10226744 (A/G) was detected in FarmCPU, MLMM, and MLM models, and a closely located SNP 27.69 kb upstream was detected in the BLINK model. This was the only significant SNP identified in the MLMM model with a PVE of 24.49%. Another SNP, S3_37752706 (C/T), was detected in FarmCPU and BLINK models.

**Table 2:** Significant SNPs identified in multiple GWAS models (FarmCPU, Blink, MLMM, and MLM) for young fruit resistance in the cucumber core collection.

SNP	Chr	Position (bp) ^1^	p-value/PVE (percent phenotypic variation explained) (%)			
			FarmCPU	BLINK	MLMM	MLM
S1_21117743	1	21,117,743	–	5.15E-13/9.01	–	1.73E-8/7.74
S2_10199046	2	10,199,046	–	8.31E-10/3.63	–	–
S2_10226744	2	10,226,744	3.85E-5/3.60	–	9.76E-10/24.49	5.95E-9/1.44
S3_18480786	3	18,480,786	1.06E-8/0.38	–	–	–
S3_37752706	3	37,752,706	3.37E-11/1.53	4.49E-10/2.09	–	–
S4_8024257	4	8,024,257	–	2.64E-9/2.25	–	–
S5_23563699	5	23,563,699	1.73E-8/3.23	–	–	–
S6_29086459	6	29,086,459	2.70E-8/2.03	–	–	–
S6_29175300	6	29,175,300	3.31E-11/5.48	–	–	–
S7_3391182	7	3,391,182	9.52E-9/0.79	–	–	–
S7_17739386	7	17,739,386	–	6.04E-9/1.35	–	–

^1^Genomic locations are according to Gy14 v. 2.1 (CuGenDB v.2; http://cucurbitgenomics.org/v2/).

Phenotypes were significantly different between accessions carrying homozygous reference vs. alternate alleles for all significant SNPs except S3_18480786 (**Supplementary Figure 2**). Of the nine SNPs, five alternate alleles led to increased resistance (lower disease scores). Of those, only two of the alternate alleles were present in ‘A4-3’ (SNP S1_21117743 and S3_37752706), suggesting that the other alleles identified by GWAS may provide additional sources of resistance. When the alternate alleles associated with lower disease scores were rare in the core collection (< 10%, i.e., < 38 accessions), the majority of accessions (64%-81%) carrying the alternate allele originated from the India/South Asia region (e.g., S1_21117743, S5_23563699, and S6_29175300) (**Table 3**). Conversely, four of the five SNPs associated with increased resistance (S1_21117743, S4_8024257, S5_23563699, and S6_29175300) were very uncommon in the East Asian accessions (0-3%). For S2_10226744, where the rare alternate allele was associated with increased susceptibility, 77% of the accessions were from East Asia. When the alternate alleles occurred frequently in the germplasm (>50%) (e.g., S3_37752706 and S7_3391182), the origins were widely distributed across regions.

**Table 3:** Geographical origin of accessions carrying the alternate alleles for the significant SNPs for young fruit resistance to *P. capsici* as identified by GWAS.

SNP	Effect^b^	Region of origin^a^								Total
		Africa	Europe	East Asia	Central/West Asia	India/South Asia	North America	Turkey	Other	
**S1_2111743**	↓	–	–	–	2	9	1	2	–	14
**S3_37752706**	↓	7	28	82	13	24	13	30	0	197
**S4_8024257**	↓	4	24	1	11	18	4	32	2	96
**S5_23563699**	↓	–	1	1	1	26	3	–	–	32
**S6_29175300**	↓	–	2	1	–	27	4	–	–	34
**S3_18480786**	–	1	2	23	2	5	1	–	–	34
**S2_10226744**	↑	1	2	23	–	2	2	–	–	30
**S7_3391182**	↑	5	43	79	30	47	37	32	2	275
**S7_17739386**	↑	1	17	17	7	15	10	17	–	84

a The regions are as defined in Wang et al. (2018).

b The effect of alternative alleles compared to reference alleles in disease score. ↓ - decreased disease score (more resistant); ↑ - increased disease score (more susceptible).

#### 3.2.2 XP-GWAS of the extreme phenotype bulks

Precise phenotyping is crucial to identify QTL associated with traits of interest, especially for quantitative traits composed of multiple small effect QTL. However, depending on the trait, phenotyping can be expensive, especially when screening a diversity panel with many lines. To determine reproducibility of QTL identified from GWAS and increase replication and accuracy of phenotyping, we used an XP-GWAS approach (Yang et al., 2015). The accessions with extreme resistant or susceptible phenotypes (29 accessions in each bulk) were retested for additional phenotyping in 2022.

**Figure 7:**
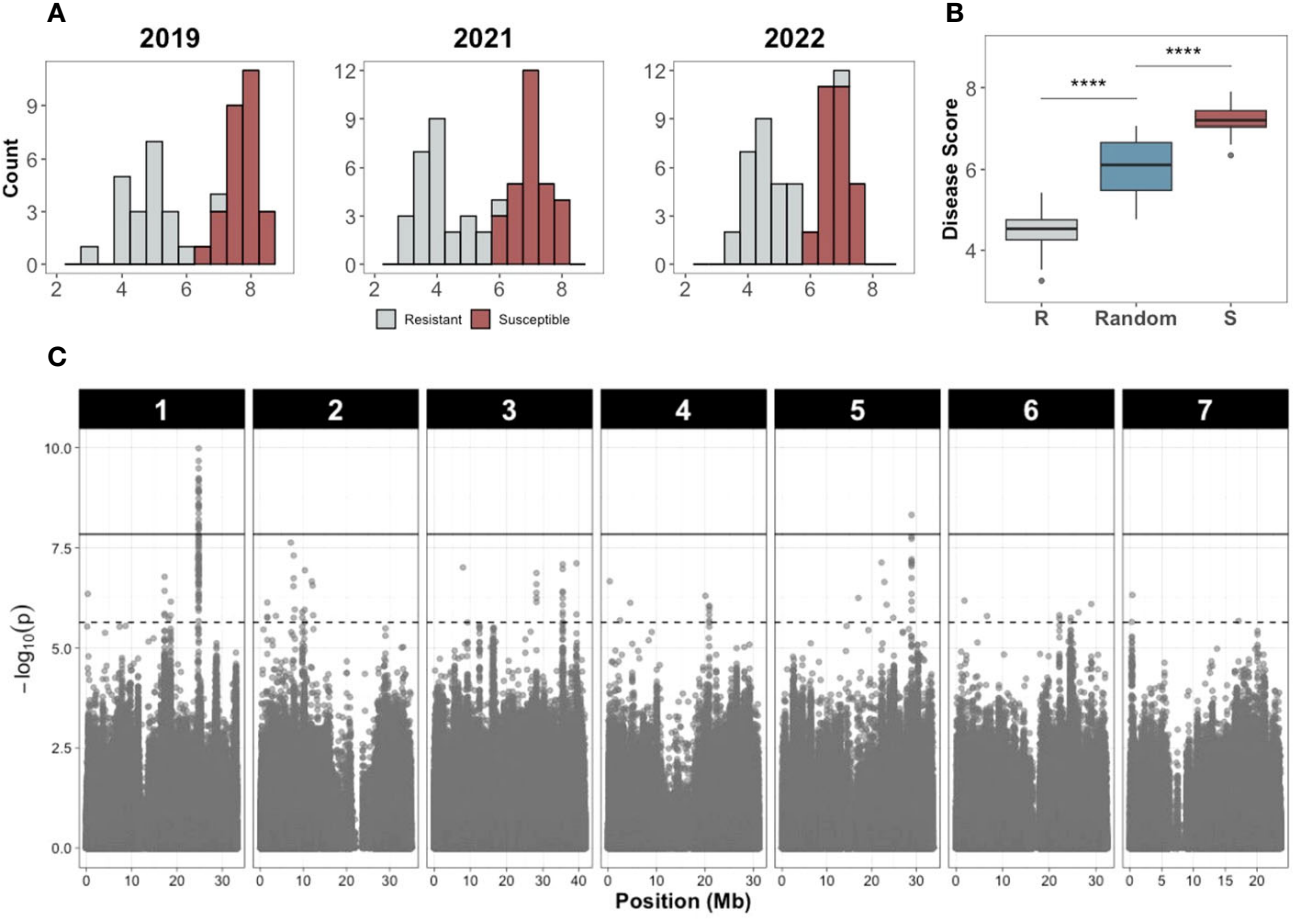
Disease score distribution and Manhattan plot of the XP-GWAS analysis to identify SNPs associated with young fruit resistance. **(A)** Disease score distribution of the resistant and susceptible bulks in different years. **(B)** Disease score values of the resistant (R), susceptible (S), and random bulks (**** indicates P<0.0001, Wilcoxon test). **(C)** Manhattan plot of the XP-GWAS analysis. The dashed line indicates the 5% FDR threshold; the solid line indicates significance threshold of Bonferroni-corrected *P* value of 0.05.

The disease score distributions of the resistant and susceptible bulks showed clear differences in multiple years (**Figure 7A**) and was reproduced in the replicated trial in 2022 (**Figure 7B**), verifying accuracy of the bulk selection for XP-GWAS analysis. Correlations for the selected resistant and susceptible bulks among 2019, 2021, 2022 were 0.755-0.912. SNP data from the selected accessions were combined via *in-silico* bulking as described in methods. XP-GWAS analysis identified 165 significant SNPs (5% FDR threshold) distributed across the seven chromosomes. The 39 significant SNPs based on the Bonferroni corrected p=0.05 threshold were located on chromosomes 1 and 5 (**Figure 7C**; **Supplementary Table 3**). The XP-GWAS SNP identified on chromosome 5 overlapped with the QTL identified by QTL-seq.

### 3.3 Identification of QTL for age-related resistance

#### 3.3.1 QTL-seq analysis

**Figure 8:**
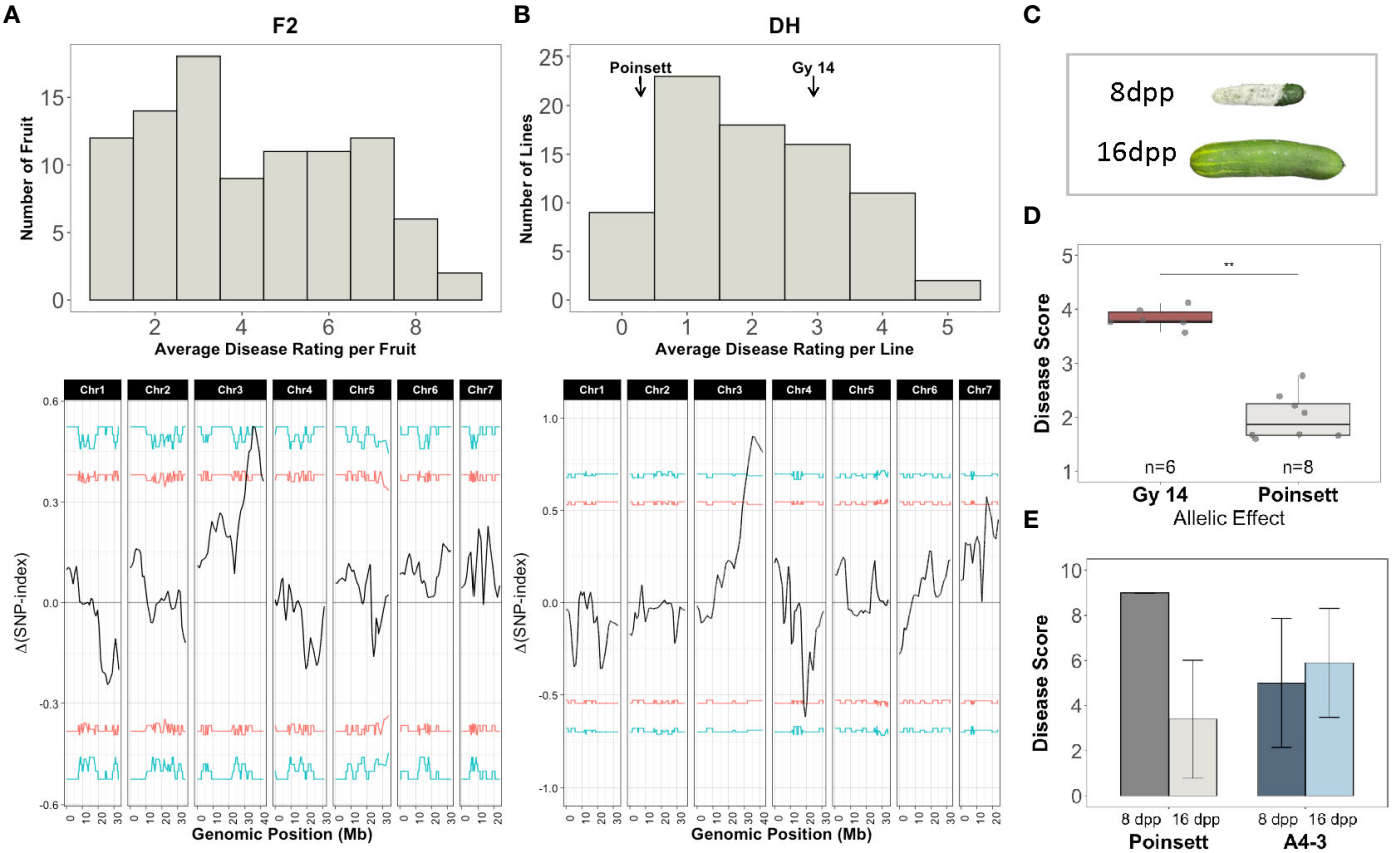
QTL-seq analysis for age-related resistance (ARR) to Phytophthora fruit rot using segregating populations derived from ‘Gy14’ (ARR-) × ‘Poinsett 76’ (ARR+). **(A)** Top: Disease rating distributions for F_2_ individuals (n=95). All fruit were harvested at 16-18 dpp. Disease rating on a 1-9 scale. Fruit were scored at 10 dpi. The 15 most resistant and susceptible individuals were selected for QTL-seq. Bottom: QTL-seq analysis. Δ(SNP-index) was calculated with a window size of 2Mb. Horizontal lines represent confidence thresholds of 95% (red) and 99% (blue). **(B)** Top: Disease rating distributions for doubled haploid lines (n=79, 3-5 fruit/line). Disease ranking on 0-5 scale. Fruit were scored at 7 dpi. The 8 most resistant and susceptible lines were selected for QTL-seq. Bottom: QTL-seq analysis. Δ(SNP-index) was calculated with a window size of 2Mb. Allele frequencies for the resistant and susceptible bulks are provided in **Supplementary Figure 1**. **(C)** Phenotype of ‘Poinsett 76’ fruit at 8 and 16 dpp photographed at 7 days post inoculation (dpi). **(D)** Mean disease ratings of F4 families (5 plants/F_4_ family) homozygous for the ‘Gy14’ or ‘Poinsett 76’ allele within the QTL on chromosome 3. **(E)** Disease ratings for ‘Poinsett 76’ and ‘A4-3’ fruit harvested at 8 and 16 dpp and scored at 7 dpi. Each value is the mean of 8-14 fruit.

Screening for ARR was performed on two populations: an F_2_ population from ‘Gy14’ (ARR-) (ARR+) (**Figure 8A**); and DH lines produced from F_1_ seed of ‘Gy14’ × ‘Poinsett 76’ (**Figure 8B**). Plants were grown in the greenhouse and a single, hand-pollinated fruit per plant was harvested at 16-18 dpp. The 15 most resistant and susceptible F_2_ individuals were selected for QTL-seq analysis (mean disease ratings of 1.32 and 7.88 for resistant and susceptible bulks, respectively; 1-9 scale). Disease scores for fruit from the 79 DH lines (3-5 fruit/line) showed high within-line variability for lines showing intermediate susceptibility; fruit from the most resistant and susceptible lines responded consistently with a mean rating of 0 and 4.8, respectively (scale 0-5) (**Figure 8C**; **Supplementary Figure 3A**). Seed from the 15 most resistant and susceptible DH lines were regrown and fruit were phenotyped in a second screen (**Supplementary Figure 3B**). The eight lines with the most consistent resistant and susceptible disease ratings in both screens were selected.

DNA from the 15 most resistant and susceptible F_2_ individuals and the 8 most resistant and susceptible DH lines were pooled for QTL-seq bulk segregant analysis. A total of 72,699 and 92,607 filtered SNPs were used in the F_2_ and DH analysis, respectively. Both analyses identified a major locus associated with resistance on chromosome 3, *qPARR3.1* (*Phytophthora ARR 3.1)* located at 34.62-38.07 and 31.08-41.68Mb, respectively (**Figures 8A, B**). To verify the QTL, KASP markers flanking the QTL on chromosome 3 were used to genotype 768 F_2_ seedlings and individuals homozygous for ‘Gy14’ or ‘Poinsett 76’ within the QTL region were self-pollinated to produce F_4_ lines. Phenotyping of fruit from a replicated trial (5 plants/F_4_ family) verified a strong effect of the QTL (**Figure 8D**).

#### 3.3.2 Identification of genes of interest within the linked locus

**Figure 9:**
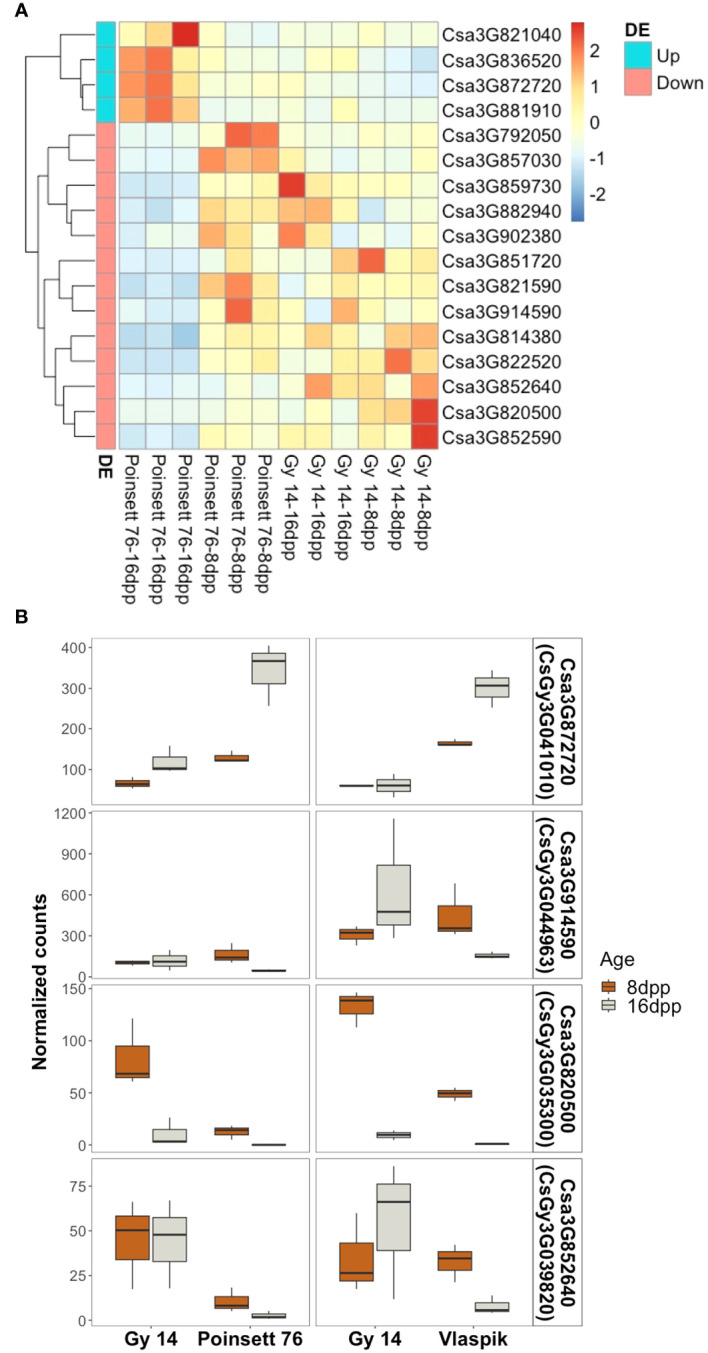
Identification of genes uniquely expressed in resistant fruit. **(A)** Heatmap of genes within the locus identified as uniquely differentially expressed in resistant-aged (16 dpp) ‘Poinsett 76’ fruit. Row clustering was based on Euclidean distances. Heatmaps are scaled by row and indicate deviation relative to mean expression across all samples. Gene names are according to Chinese Long v.2 (CuGenDB; http://cucurbitgenomics.org/). (Read count data and corresponding Gy14v.2.1 gene names are provided in **Supplemental Table 4**). **(B)** Genes uniquely up- or down-regulated in transcriptome comparisons with two ARR+ genotypes (‘Poinsett 76’ and ‘Vlaspik’). Boxplots show the distribution and median (dark line) of normalized read counts from two experiments. ‘Gy 14’ is susceptible at both ages (8 and 16 dpp); ‘Poinsett 76’ and ‘Vlaspik’ are susceptible at 8 dpp and become resistant at 16 dpp. Three biological replicates of each age-genotype combination were used in each experiment.

Transcriptome analysis of the parental lines at 8 and 16 dpp was used to identify genes of interest within the region of *qPARR3.1*. ARR results from developmental changes that occur prior to inoculation, either as a result of production of preformed resistance mechanisms, or a change in capacity to rapidly respond to infection (Mansfeld et al., 2017; Mansfeld et al., 2020). Therefore, we sought to identify developmental changes in gene expression that are unique to cultigens that become resistant vs. those that remain susceptible. Genes were considered of interest if they showed differential expression with age in ‘Poinsett 76’ *and* were also differentially expressed in ‘Poinsett 76’ vs. ‘Gy14’ at 16dpp. Of the 1,240 annotated genes in this region (CL9930v2; CuGenDB), only four genes were uniquely upregulated in resistant ‘Poinsett 76’ (i.e., up in ‘Poinsett 76’ fruit peels at 16 dpp vs. ‘Poinsett’ 76 at 8 dpp, and in ‘Poinsett 76’ at 16 dpp vs. ‘Gy14’ at 16dpp). Thirteen genes were uniquely downregulated in resistant peels (down in ‘Poinsett 76’ fruit peels at 16 dpp vs. ‘Poinsett 76’ at 8 dpp, and in ‘Poinsett 76’ at 16 dpp vs. ‘Gy14’ at 16dpp) (**Figure 9A**; **Supplementary Table 4**).

To further examine genes potentially contributing to ARR, we screened our previously published transcriptome data from developing ‘Vlaspik’ fruit (ARR+) compared to ‘Gy 14’ fruit (ARR-) (Mansfeld et al., 2017) to identify genes uniquely up- or downregulated in both ARR+ cultivars at 16 dpp. Of the 17 genes identified above, one upregulated gene and three downregulated genes had similar expression patterns in ‘Poinsett 76’ and ‘Vlaspik’ (**Figure 9B**). The up-regulated gene, *Csa3G872720 (CsGy3G041010 in Gy14 v.2.1)*, showed consistently significantly higher expression in resistant ‘Poinsett 76’ and ‘Vlaspik’ at 16 dpp. Expression in 16 dpp ‘Poinsett 76’ fruit was >2.5-fold higher compared to 8 dpp susceptible ‘Poinsett 76’ fruit and susceptible ‘Gy 14’ 16 dpp fruit. Similarly, expression in resistant ‘Vlaspik’ fruit was greater than 4-fold higher when compared to susceptible ‘Gy14’ 16 dpp fruit, and 1.8-fold higher when compared to susceptible ‘Vlaspik’ 8 dpp fruit. In contrast, while the downregulated genes had statistically lower values in resistant fruit in both genotype comparisons, the patterns observed were not obviously consistent with our model of ARR; i.e., distinctly different expression levels in 16 dpp resistant fruit compared to the susceptible samples.

*CsGy3G041010* located at 38,365,049-38,373,217 bp (Gy14 v. 2.1), is annotated to encode a putative RING-type E3 ubiquitin transferase, a U-box domain and WD40 repeat containing protein. Sequence comparisons from 2 kb upstream until the end of its 3’UTR in ‘Gy 14’ (ARR-), ‘Poinsett 76’ (ARR+) and ‘Vlaspik’ (ARR+) identified 15 SNPs and 5 INDELs (**Supplementary Table 5**) that differed between ‘Gy14’ and ‘Poinsett 76’. Four of the variants were within 2kb upstream of the transcription start site; eight were in introns, one of which was close to a splice site; three were in the 3’UTR. Six SNPs were in exons, five of which cause non-synonymous amino acid changes. ‘Vlaspik’, which is a commercial F_1_ hybrid, was heterozygous at all variants. In contrast to ‘Poinsett 76’ which shows a strong drop in disease score for 16 dpp vs. 8 dpp fruit, the disease rating of ‘A4-3’ remained essentially constant at 8 dpp vs. 16 dpp indicating the distinct nature of ARR and young fruit resistance in these genotypes (**Figure 8E**). The *CsGy3G041010* allele in ‘A4-3’ matches that of ‘Gy14’.

## 4 Discussion

### 4.1 Employing multiple strategies to detect QTL for resistance to Phytophthora fruit rot

The types of populations most frequently used to identify QTL associated with traits of interest are segregating biparental populations and genetically variable natural populations. In this work, both types of populations were used to search for QTL associated with young fruit resistance to Phytophthora fruit rot in cucumber. QTL-seq, a bulk segregant analysis (BSA) approach performed on progeny from biparental populations that express extreme phenotypes, provides a quick and specific way to detect QTL. Furthermore, using different population structures such as F_2,_ RIL, and DH can provide additional power for QTL detection. QTL-seq was performed on both young fruit resistance and ARR. The analyses discovered three QTL associated with the young fruit resistance and one for ARR. For young fruit resistance, the QTL were located on chromosomes 1, 5, and 6; the strongest effect was from the QTL on chromosome 5, *qPFR5.1*. For ARR, which appears to have a major gene component, a QTL at the end of chromosome 3 was identified. The ability to identify QTL and the size of the genomic region identified is limited to the genetic variation between the two parents and is influenced by population structure and size (Mackay and Caligari, 2000; Li and Xu, 2022). The lengths of QTL from QTL-seq can be large, for example, *qPFR5.1* was ~7 Mb and *qPARR3.1* was ~10 Mb. To narrow the QTL for young fruit resistance, screening of additional RIL and F_3_ populations that were enriched for recombination within the region refined *qPFR5.1* to ~1 Mb.

The second approach, use of a diversity panel such as the cucumber core collection, provides the opportunity to identify additional QTL associated with the resistances in a germplasm with higher genotypic diversity. The main drawbacks of using a diversity panel include higher expenses to phenotype and genotype the large population size, and the difficulty to detect rare alleles associated with the traits (Alqudah et al., 2020; Uffelmann et al., 2021). In addition to traditional GWAS, where the whole collection is phenotyped and genotyped, a second method of association analysis, extreme-phenotype genome-wide association study (XP-GWAS) can be used to reduce experimental costs by reducing the number of entries to be included (Yang et al., 2015). XP-GWAS is typically used to reduce sequencing costs and has been applied to several crops with different target traits. Some recent examples include rice (Xiao et al., 2017), apple (Kumar et al., 2022), switchgrass (Poudel et al., 2021), sugar beet (Ries et al., 2016), and wheatgrass (Crain et al., 2023). In this case, however, as sequence data was already available for the full collection (Yu et al., 2023), we were able to use XP-GWAS to reduce phenotyping costs associated with additional replications. By focusing on the lines with extreme phenotypes, the rare alleles associated with resistance can be enriched within the bulk and are more likely to be detected (Yang et al., 2015; Zou et al., 2016). Although performing XP-GWAS still requires known phenotypes of each line, once candidates for the extremes are identified, the subsequent phenotyping expense for replication can be reduced. In this research a preliminary screening of the complete cucumber core collection was performed from 2019-2021. To verify the phenotypes, selected accessions were grown in the following year with additional replications to increase the accuracy of phenotyping.

It should be noted that when performing association analysis, the same data sets analyzed using different programs can give somewhat different results due to the default assumptions written within each software. As a result, peak SNPs may be offset by several Mb. For example, the significant SNP on chromosome 3 detected in rMVP (Yin et al., 2021), another widely used R-based GWAS software, was about 3 Mb away from the significant SNP detected on chromosome 3 in GAPIT3.0 (34,803,363 vs. 37,752,706, respectively). Therefore, replication and comparison among tools and models are recommended to avoid false positives (Chanock et al., 2007). Similarly, different experiments with standard QTL or QTL-seq analyses can give somewhat different estimates of QTL location (e.g., powdery mildew and downy mildew resistance QTL (Wang et al., 2020). In other cases, QTL may be somewhat complex, composed of more than one contributing factor as was observed for cucumber downy mildew (Berg et al., 2020), possibly contributing to different assessments of QTL location. These observations can have implications for consideration of appropriate regions for introgression of disease resistance QTL.

**Figure 10:**
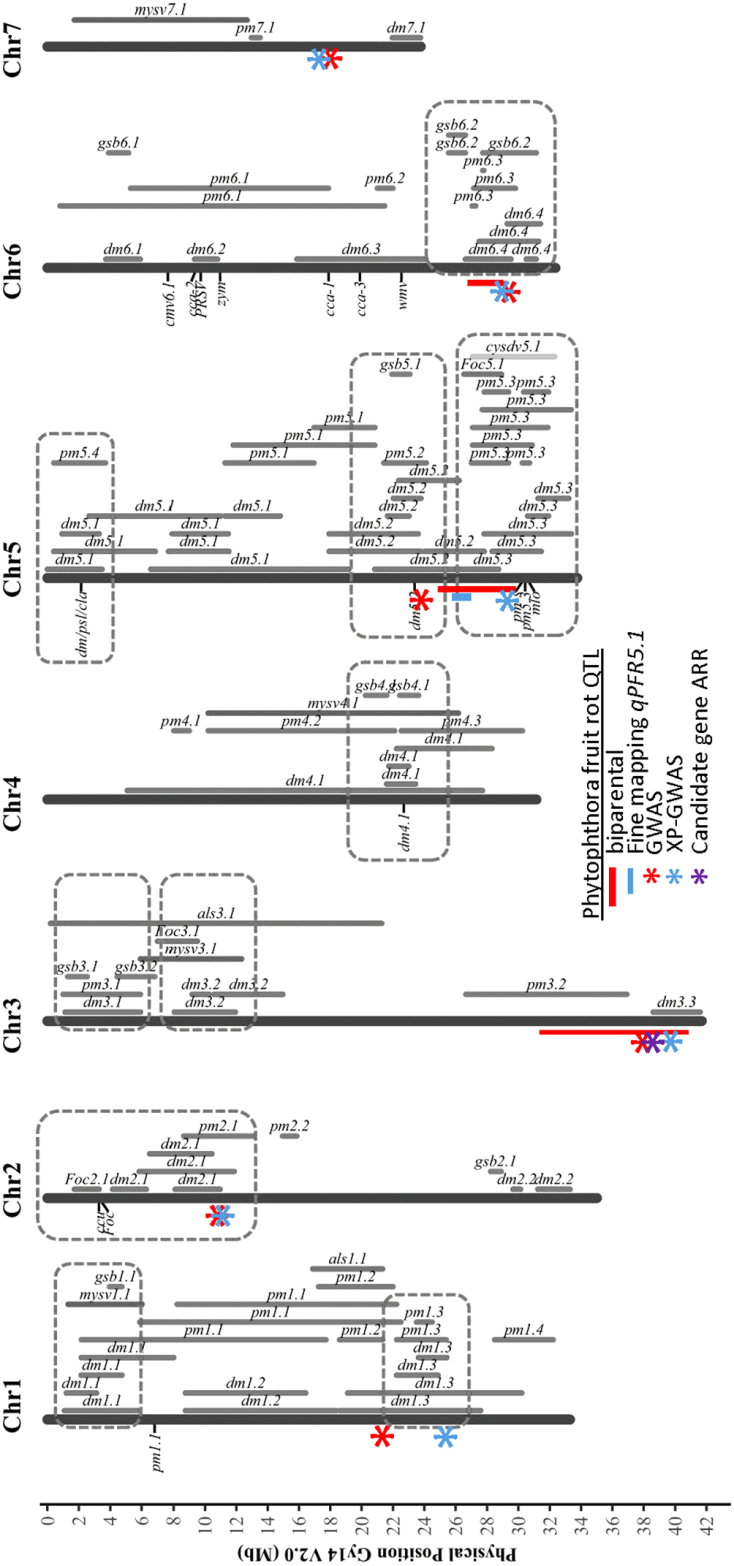
Chromosomal locations of QTL identified for Phythophthora fruit rot of cucumber in relation to prior QTL identified for resistances to other cucumber diseases. The indicated PFR QTL were identified from multiple analyses: red bar – biparental QTL-seq; blue bar – fine mapping of biparental populations; red asterisk GWAS of cucumber core collection; blue asterisk XP-GWAS; purple asterisk – candidate gene identified by RNAseq analyses. Figure is adapted from Wang et al., 2020 *Horticulture Research* under Creative Commons license http://creativecommons.org/licenses/by/4.0/. DM, downy mildew; PM, powdery mildew; ALS, angular leaf spot; Foc, fusarium wilt; GSB, gummy stem blight; MYSV, melon yellow spot virus; CYSDV, cucurbit yellow stunting disorder virus.

Using multiple approaches, several SNPs significantly associated with resistance to Phytophthora fruit rot were identified in closely-located positions, giving greater confidence to their contributions (**Figure 10**). In our results, regions were identified by more than one approach on chromosomes 1, 2, 3, 5, 6, and 7. The QTL peaks detected on chromosome 1 were located at 21.17 Mb by GWAS and 24.75 Mb by XP-GWAS. The QTL on chromosomes 5 and 6 were consistently identified by QTL-seq, GWAS and XP-GWAS methods (on chromosome 5 all were located within 7 Mb, and on chromosome 6 all were within 3 Mb). On chromosome 3, the peak SNP was located at 37.75 Mb in GWAS and at 39.29 Mb in XP-GWAS. Both fall within the QTL region previously identified from QTL-seq analysis for ARR including the candidate gene *CsGy3G041010* located at 38.36 Mb. In most cases, the QTL identified for Phytophthora fruit rot also coincided with previously identified QTL for other diseases.

The identification of multiple QTL for young fruit resistance is consistent with quantitative traits consisting of multiple small effect QTL. Studies identifying QTL for resistance to *P. capsici* in other species also have indicated polygenic architecture, including several examples in pepper (*Capsicum annum*) and squash (*Cucurbita pepo* and *Cucurbita moschata)* (Barchenger et al., 2018; Ramos et al., 2020; Vogel et al., 2022). The signal on chromosome 5 was stronger in QTL-seq and XP-GWAS compared to GWAS, possibly due to the use of the resistant line ‘A4-3’ in the bi-parental QTL-seq analysis, and the enrichment of rare alleles in the resistant bulk for XP-GWAS analysis.

Although the SNPs identified from the association analyses in this study were screened for young fruit resistance, *qPARR3*.1 from QTL-seq analysis was also detected by GWAS and XP-GWAS analyses. This may result from closely located genes within the *qPARR3*.1 region that confer young fruit resistance. It may also be possible that for some accessions the ARR-associated QTL may function earlier during fruit development and contribute to young fruit resistance. A BLAST search (Swiss-Prot database) of the protein sequence for the candidate gene for ARR on chromosome 3 reveals that this gene is a homologue of the *LIN* gene found in *Medicago truncatula* (E = 0e0, Identity = 58.38%, Positives = 72.86%). In *M. truncatula* LIN functions in early rhizobial symbiotic nodule formation and mutation of LIN leads to a suppression of nodule development (Kiss et al., 2009). *LIN* was shown to not be required for nodule organogenesis, however *LIN* expression was associated with rhizobial root infection (Kiss et al., 2009). Interestingly, while other rhizobial-symbiosis mutants were more resistant to infection, the *lin-2* mutant was shown to be extremely susceptible to the pathogen *Phytophthora palmivora* (Rey et al., 2015). It has been hypothesized that the protein modulates defense responses by means of its U-box domain and the ubiquitination of target proteins (Kiss et al., 2009).

In addition to the QTL identified by QTL-seq in biparental populations, novel SNPs were also discovered by association analyses, providing potential additional resources for future breeding efforts. Multiple alternative alleles of the significant SNPs leading to stronger resistance were found in accessions originating from India and South Asia, the primary and secondary centers of origins of cucumber (Lv et al., 2012; McCreight et al., 2013). During the process of subsequent domestication and dissemination, cucumber germplasm diverged between East Asia vs. Eurasia and the West (Europe, Africa, North America) (Qi et al., 2013; Wang et al., 2018). This divergence, which is evident in fruit morphology, is also reflected in genetic composition, showing differentiation of cucumber into three major phylogenetic clades: India/South Asia, East Asia, and the West (Qi et al., 2013; Wang et al., 2018; Grumet et al., 2021). Consistent with the resistance-associated alleles identified in this study, prior screening indicated that the accessions that were resistant to Phytophthora fruit rot were mainly from India, especially North and Central India (Colle et al., 2014; Grumet et al., 2020). Although there were also resistant accessions that originated from East Asia, alleles associated with higher susceptibility were more frequently traced to East Asian accessions.

### 4.2 QTL hotspots for disease resistance

Numerous studies have been conducted to identify QTL of various disease resistances in cucumber as summarized in a recent review by Wang et al. (2020). Collectively these studies implied the presence of disease resistance gene/QTL hot spots on several chromosomes. Interestingly, most of the QTL and significant SNPs identified here for Phytophthora fruit rot also co-localized with the hot spots including resistances to downy mildew, powdery mildew, fusarium wilt, and gummy stem blight (**Figure 10**). The aggregation of these QTL suggests that these genomic regions play an important role in disease resistance to fungal and oomycete pathogens. Oomycete and fungi are two evolutionary distinct groups; however, they share similar strategies in terms of infection, e.g., specialized infection structures such as appressoria, infection hyphae and haustoria, and secreting cell-wall-degrading enzymes to facilitate cell wall penetration (Latijnhouwers et al., 2003). Though the mechanisms of resistances to these pathogens remain unknown, similar or the same defense-associated genes might be activated in response to infection and result in clusters of disease resistance QTL.

In addition to *Phythophora* the other major disease leading to severe loss of pickling cucumber production in midwestern United States is downy mildew caused by *Pseudoperonospora cubensis* (Hausbeck and Lamour, 2004; Savory et al., 2011). Toward the end of chromosome 5 there are two disease resistance QTL clusters including resistances to powdery mildew and downy mildew pathogens. *qPFR5.1* for young fruit resistance identified in this work is located in one of these clusters and is adjacent to two downy mildew resistance QTL, *dm5.2* and *dm5.3* (Wang et al., 2016; Tan et al., 2022). The narrowed QTL identified by fine mapping allowed us to distinguish the boundaries between these three QTL. With distinct borders between the QTL, molecular markers targeting specific QTL can be designed for marker assisted selection, and to develop breeding lines that can confer multiple diseases by pyramiding resistance QTL from different genetic backgrounds.

## 5 Conclusions

Resistance to *Phytophthora capsici* is an agronomically important trait in cucumber but currently no resistant commercial varieties are available due to the limited research and intricate genetic natures of both the pathogen and the host. A combination of approaches of QTL-seq and associated analyses were used to identify QTL for resistances to Phytophthora fruit rot in cucumber. Multiple QTL were identified for young fruit resistance. The largest effect QTL, *qPFR5.1*, was located on chromosome 5, and narrowed to approximately 1 Mb. A major effect QTL for ARR, *qPARR3.1*, was found at the end of chromosome 3, and a candidate gene identified from comparative transcriptomic analyses of cucumber peels. Additional SNPs associated with resistance were discovered from GWAS and XP-GWAS analyses of the USDA cucumber core collection. The close vicinity of the QTL and SNPs identified from multiple analyses strengthened the credibility of these findings. Several of the findings also corresponded with previously identified disease resistant hot spots in cucumber. Collectively, the results of this work can provide useful information for future studies to understand mechanisms of resistance to *P. capsici* in cucumber and breed for varieties with resistance to Phytophthora fruit rot.

## Data availability statement

The datasets presented in this study can be found in online repositories. The names of the repository/repositories and accession number(s) can be found below: PRJNA1007410 and PRJNA1006651 (SRA).

## Author contributions

YL: Conceptualization, Data curation, Formal Analysis, Investigation, Methodology, Writing – original draft, Writing – review & editing. BM: Conceptualization, Data curation, Formal Analysis, Investigation, Writing – original draft, Writing – review & editing. XT: Data curation, Formal Analysis, Writing – review & editing. MC: Investigation, Methodology, Writing – review & editing. FC: Resources, Writing – review & editing. YW: Funding acquisition, Resources, Writing – review & editing. ZF: Data curation, Formal Analysis, Funding acquisition, Writing – review & editing. RG: Conceptualization, Formal Analysis, Funding acquisition, Project administration, Writing – original draft, Writing – review & editing.
