# Supplementary material for: Identification of QTL associated with resistance to Phytophthora fruit rot in cucumber (Cucumis sativus L.)
Source: Front Plant Sci. 2023 Nov 15;14:1281755. doi: 10.3389/fpls.2023.1281755 (PMC10693349; doi:10.3389/fpls.2023.1281755)
Supplement: Supplementary file 1 [file Presentation_1.pptx]

## Slide 1
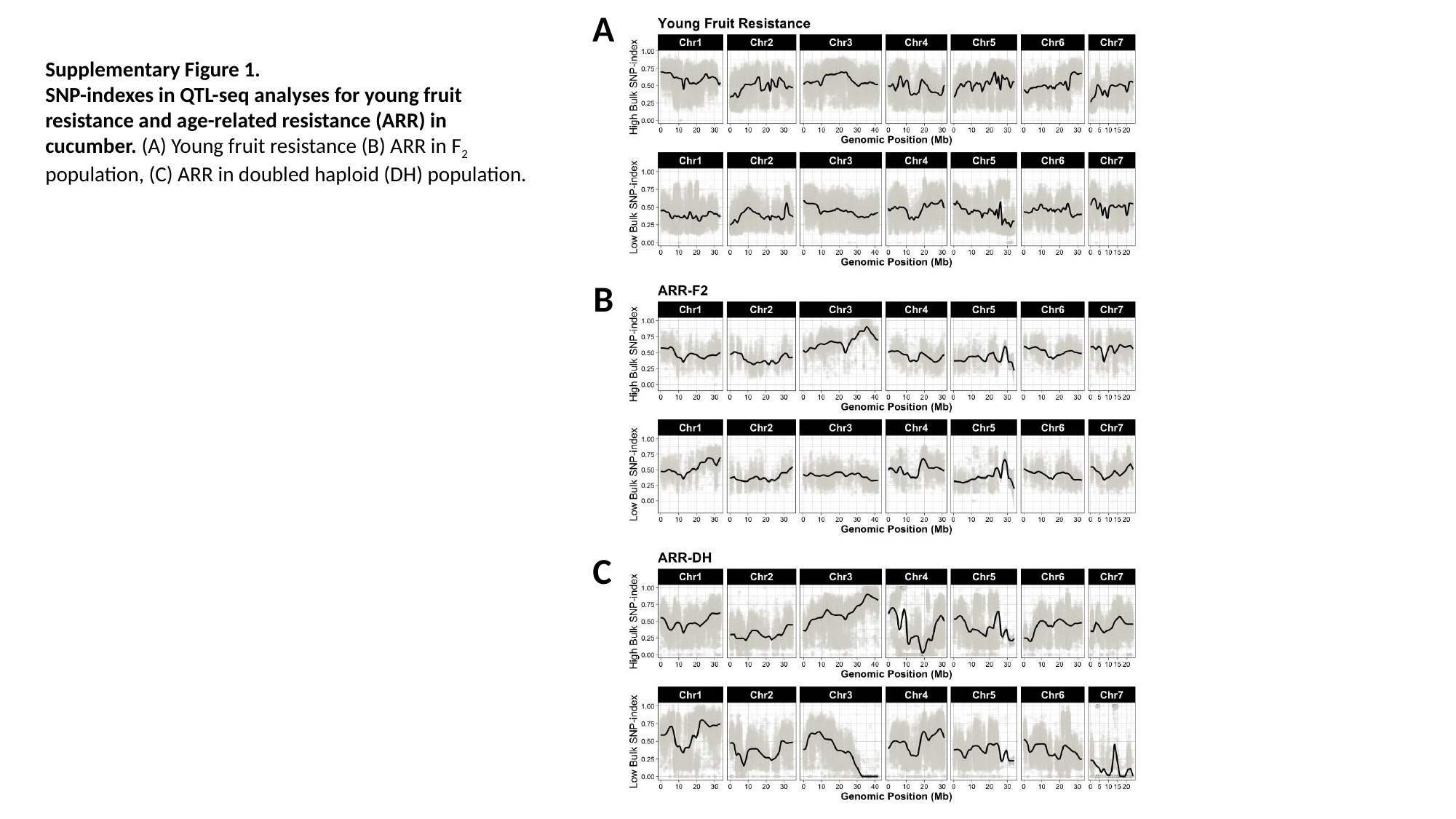

A
Supplementary Figure 1.
SNP-indexes in QTL-seq analyses for young fruit resistance and age-related resistance (ARR) in cucumber. (A) Young fruit resistance (B) ARR in F2 population, (C) ARR in doubled haploid (DH) population.
B
C

## Slide 2
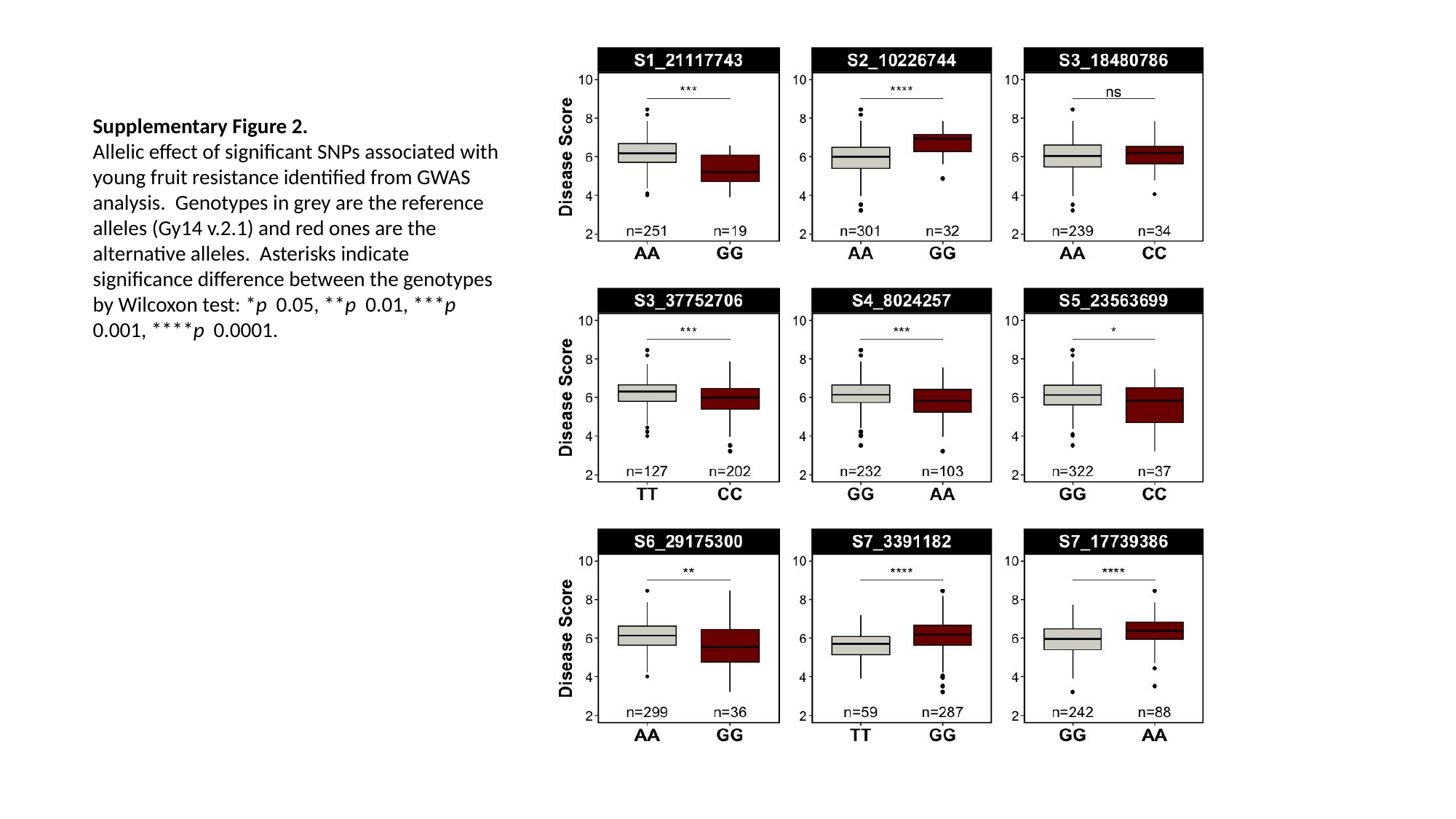

## Slide 3
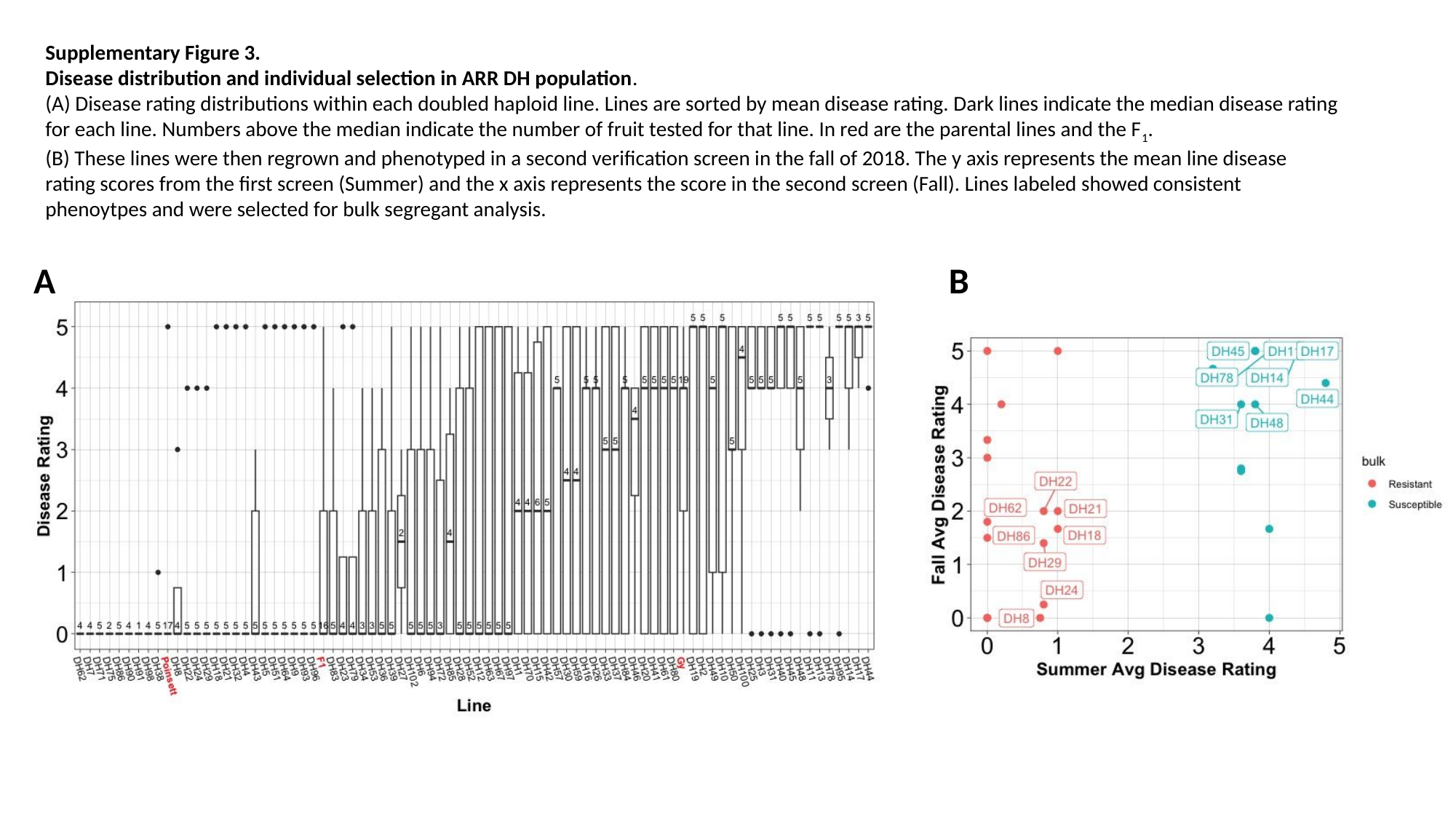

Supplementary Figure 3.
Disease distribution and individual selection in ARR DH population.
(A) Disease rating distributions within each doubled haploid line. Lines are sorted by mean disease rating. Dark lines indicate the median disease rating for each line. Numbers above the median indicate the number of fruit tested for that line. In red are the parental lines and the F1.
(B) These lines were then regrown and phenotyped in a second verification screen in the fall of 2018. The y axis represents the mean line disease rating scores from the first screen (Summer) and the x axis represents the score in the second screen (Fall). Lines labeled showed consistent phenoytpes and were selected for bulk segregant analysis.
A
B
